# Refractive errors in mixed breed dogs of different ages

**DOI:** 10.17221/106/2021-VETMED

**Published:** 2023-01-10

**Authors:** Agnieszka Balicka, Jowita Zwolska, Mateusz Szadkowski, Alexandra Trbolova, Ireneusz Balicki

**Affiliations:** ^1^Small Animals Clinic, University of Veterinary Medicine and Pharmacy in Košice, Košice, Slovak Republic; ^2^Department and Clinic of Animal Surgery, Faculty of Veterinary Medicine, University of Life Sciences in Lublin, Lublin, Poland

**Keywords:** autorefractor, canine, emmetropia, hyperopia, myopia

## Abstract

The purpose of the study was to evaluate the occurrence and range of refractive errors in dogs of different ages. A total of 99 clinically healthy, mixed-breed mesocephalic dogs were included in the study and divided into three different age groups according to the current human/pet analogy chart: 40 adults (23 males, 17 females, 1–8 years old, 3–70 kg), 21 seniors (14 males, 7 females, 6–11 years old, 7–42 kg), and 38 geriatrics (22 males, 16 females, 8–13 years old, 5–45 kg). All the dogs underwent an ophthalmic examination, including Schirmer tear test, tonometry, biomicroscopy, and ophthalmoscopy. Neither eye drops nor pharmacological sedatives were administered before the autorefractometry. The refractive states were assessed bilaterally using a hand-held Retinomax 3 (Righton) autorefractor. The results underwent statistical analysis using Statistica v12 software (ANOVA and *t*-test). A *P*-value < 0.05 was considered as significant. Emmetropia, defined as a refractive state > −0.5 D and < +0.5 D, was found in 36% of the adult, 43% of the senior, and 38% of the geriatric patients. Anisometropia was found in 1% of the adult, 9.5% of the senior and 5.5% of the geriatric dogs when the refractive power of the two eyes differed ≥ 1.0 myopia ≤ −0.5 D and hyperopia ≥ +0.5 D were found in 23% and 41% of the adult eye globes as well as 24% and 33% in the senior dogs and 15% and 47% in the geriatric dogs, respectively. The maximal values of the myopia in the adult and geriatric dogs were −2.5 D and −2.75 D, respectively. The maximal values of the hyperopia in the adult and geriatric dogs were 1.75 D and 2.5 D, respectively. No statistically significant correlation was found between the groups. Ametropia is a common refractive state for dogs of different ages. The most frequent refractive state in ametropic mixed-bed dogs in all age groups is hyperopia.

The optic system of the eye is composed of the cornea, aqueous humour, crystalline lens, vitreous and retina. In emmetropia, light converges on the retina to form a focused picture. In the opposite situation, called ametropia, a refractive error of the eye occurs. Refractive disorders include myopia and hyperopia, also known as near-sightedness and far-sightedness, respectively (Davidson et al. 1993; Black et al. 2008). In the case of anisometropia, the refractive power of the two eyes is unequal. In veterinary medicine, the exact difference in the dioptre has not been defined and varies between 0.5–1.0 D in the case of anisometropia (Kubai et al. 2008; Maehara et al. 2011). The refractive error may be caused by an abnormal corneal curvature, the abnormal strength of the crystalline lens or changes in the length of the optical axis of the eye (Davidson et al. 1993). The analysis of the refractive status requires an individual approach, as it is breed- and age-specific (Kubai et al. 2008). In older dogs, problems with the eyesight are usually secondary to corneal diseases, cataracts or retinal disorders (Mutti et al. 1999). Eyesight is important for interacting with the environment of animals (Itoh et al. 2011). Ageing is a physiological decline when changes in the senses, including vision, hearing, and olfaction, can be observed. In older dogs, a decreased ability to react becomes common, secondary to the physiological decline (Bellows et al. 2015).

There are several methods used in the diagnostics of refractive errors. The most popular methods used in veterinary medicine are retinoscopy and autorefractometry (Itoh et al. 2011). In retinoscopy, an examiner places the source of the light in front of the patient’s eye and checks the degree of its reflection in the patient’s retina. Then, the examiner moves the light beam along the pupil and observes the movement of the reflection, with attached lenses “reducing” the reflection of the light. An autorefractometer/autorefractor is a digital device (computer-controlled) for the automatic, quick and objective measurement of refractive errors. The method is based on using sensors that detect the reflections of infrared light from the eye.

With an increasing refractive error, the dog’s orientation decreases. In a study detecting canine behaviour in a field trial, it was revealed that dogs wearing lenses causing refractive error had problems negotiating obstacles or even gaining owners. It has also been revealed that puppies diagnosed with a refractive error require a longer time for training than puppies with emmetropia (Ofri et al. 2012). An analysis of the population of guide dogs revealed refractive errors were far less common than in other dogs. This could be caused by the strict breeding selection of animals taking animal behaviour and orientation into consideration (Kubai et al. 2008).

The aim of the study was to evaluate the occurrence and range of refractive errors in dogs of different ages.

## MATERIAL AND METHODS

This study was conducted on 99 clinically healthy mesocephalic dogs. All the dogs were divided into three groups according to the current human/pet analogy chart (Fortney 2012): 40 adults (23 males, 17 females, 1–8 years old, 3–70 kg), 21 seniors (14 males, 7 females, 6–11 years old, 7–42 kg), and 38 geriatrics (22 males, 16 females, 8–13 years old, 5–45 kg). The dogs underwent an ophthalmic examination, including a Schirmer tear test, tonometry, biomicroscopy and ophthalmoscopy. The ophthalmic examinations included a neuro-ophthalmology examination and behavioural testing of the vision [testing for the menace response, dazzle reflex, visual placing (if possible), and visual tracking, pupillary light reflex and chromatic pupillary light reflex]. All the dogs had no history of ocular abnormalities or vision impairment. The refractive status was assessed in the dogs that were not diagnosed with eye disorders or diseases. All the dogs were patients of the Department and Clinic of Animal Surgery at the University of Life Sciences in Lublin. The canine patients were privately owned pet dogs or shelter dogs. All the dogs were vaccinated and dewormed according to the schedule. Food was given to the dogs in different qualities and quantities according to the owners. Water was given *ad libitum* to all the dogs.

Before the autorefractometry, the dogs were not given any sedatives or cycloplegic eye drops. The refractive states were assessed bilaterally using a hand-held Retinomax 3 autorefractor (Righton). Emmetropia was defined as a refractive state > −0.5 D and < +0.5 D. Anisometropia was found when the refractive power of the two eyes differed ≥ 1.0 D, myopia was defined as ≤ −0.5 D and hyperopia was defined as ≥ +0.5 D. The results underwent a statistical analysis using the Statistica v12 software [analysis of variance (ANOVA) – “Kruskal-Wallis test” and unpaired *t*-test]. The statistical analysis was performed comparing all the age groups. A *P*-value < 0.05 was considered to indicate significant impairment.

The research was approved by the Scientific Research Committee of the Department and Clinic of Animal Surgery at the University of Life Sciences in Lublin (No. 3/2018) concerning non-experimental clinical patients. The owners were informed about the details of the conducted clinical trials, and they verbally gave their consent. The study was performed in accordance with Polish law and with Directive 2010/63/EU of the European Parliament and of the Council of 22 September 2010 on the Protection of Animals Used for Scientific Purposes, Chapter I, Article 1, point 5(b).

## RESULTS

**Figure 1:**
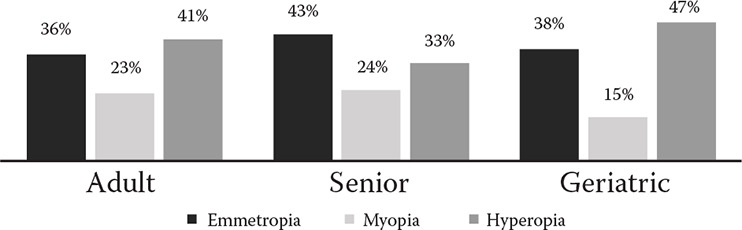
Percentage presentation of the refractive status of dogs of different ages

**Figure 2:**
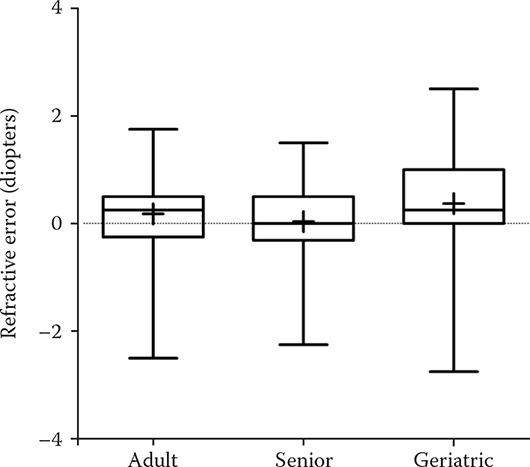
Refractive error value in dogs of different ages

The mean refractive error of all the examined dogs was 0.2 ± 0.9 D. Among the dogs of different ages, normal eyesight, in at least one eye, was diagnosed in 56.6% of dogs. Emmetropia (condition of the eye with normal refractive power), defined as > −0.5 D and < +0.5 D, was found in 36% of the adult, 43% of the senior and 38% of the geriatric eye globes. Regarding dogs with bilateral emmetropia, there were 8 (20%) adult dogs, 4 (19%) senior dogs, and 8 (21%) geriatric dogs. Anisometropia was found in 2.5% of the adult, 19% of the senior and 10.5% of the geriatric dogs. Myopia (≤ −0.5 D) and hyperopia (≥ +0.5 D) were found in 23% and 41% of the adult eye globes as well as 24% and 33% of the senior and 15% and 47% of the geriatric eye globes, respectively. All the results of the examinations of the dogs of different refractive statuses of different ages are presented in Table 1 and Figure 1. The maximal values of hyperopia in the adult, senior and geriatric dogs were 1.75 D, 1.5 D and 2.5 D, respectively. The maximal values of myopia in the adult, senior and geriatric dogs were −2.5 D, –0.25 D and −2.75 D, respectively (Figure 2). No statistically significant correlation between the adult, senior and geriatric dogs was found between the dogs with myopia. The geriatric dogs had significantly higher hyperopia than senior dogs (*P*-value 0.02).

**Table 1:** Different aged dogs with the refractive status – Number of dogs and number of analysed eye globes with emmetropia, anisometropia, and myopia, hyperopia

Refractive status		Adult	Senior	Geriatric
Dogs	emmetropia	8	4	8
	anisometropia	1	4	4
Eye globes	myopia	18	10	11
	hyperopia	33	14	36
	emmetropia	29	18	29

## DISCUSSION

Examination of the refractive status of 50 dogs of different breeds comparing the Welch Allyn SureSight^™^ autorefractor with the retinoscopy in normal dogs revealed a mean ± SD non-cycloplegic autorefraction spherical equivalent of −0.42 ± 1.13 D (Groth et al. 2013).

In our study, the mean refractive error of all the examined dogs was 0.2 ± 0.9 D. Both results are recognised in veterinary medicine as emmetropia. Hernandez et al. (2016) analysed the refractive status in nine beagle dogs between 1 and 14 years of age. In the study, myopic shifts close to –2 D and –3 D were observed with ageing (Hernandez et al. 2016). Similar to our study, the authors used autorefractors. Although, in our study, only 24% of the senior and 15% of the geriatric dogs were diagnosed with myopia, the stage of advancement of short-sightedness was the highest in the geriatric dogs gaining a level of –2.75 D. According to our results, most of the ageing dogs had far-sighted vision, 33% of the senior dogs and 47% of the geriatric dogs. Hernandez et al. (2016), referring to differences in the refractive status between studies, emphasised the size of the animals, breed, gender and age as affecting factors. Hernandez et al.’s (2016) study was conducted on 14 beagle dogs, while our study was conducted on 99 mixed-breed mesocephalic dogs. In this study, three analysed beagle dogs were diagnosed with nuclear sclerosis (one mild, one mild/moderate and one dense). A study conducted in 1992 on 240 various dogs also revealed myopia in older dogs with nuclear sclerosis of crystalline lenses (Murphy et al. 1992). None of the dogs included in authors’ study were diagnosed with nuclear sclerosis.

The refractive state of eyes of various breed dogs was described in number of publications determining specific tendencies (Murphy et al. 1992; Kubai et al. 2008; Williams et al. 2011; Groth et al. 2013; Hernandez et al. 2016; Balicka et al. 2018; de Oliveira et al. 2020).

The study assessing the refractive state of eyes in 1 440 dogs of 90 breeds revealed myopia occurring in Rottweilers, Collies, Miniature Schnauzers, and Toy Poodles. Hyperopia was the most commonly diagnosed in Australian Shepherds as well as Bouvier des Flandres and Alaskan Malamute dogs. In Kubai’s et al. (2008) study, the mean refractive error was –0.05 ± 1.36 D. The study revealed that the degree of myopia develops with age. Our study was conducted on 99 dogs of mixed breeds and different sizes. In our study, the mean refractive error of all the examined dogs was 0.2 ± 0.9 D. Although the highest value of myopia, at the level of –2.75 D, was diagnosed in the geriatric dogs in our study, most ageing dogs had hyperopia. The summary of the results of the study and the conclusions of the others’ research projects are presented in Table 2.

**Table 2:** Comparison of the results of the studies regarding the refractive status of the dogs with different ages

Detected refractive error	References		Results of our study
Mean refractive error in dogs	−0.42 ± 1.13 D; 50 different breed dogs (Groth et al. 2013)	–0.05 ± 1.36 D; 1 440 dogs of 90 different breeds (Kubai et al. 2008)	0.2 ± 0.9 D; 99 mixed breed dogs
Refractive error in senior dogs	myopia – exanimated 2 seniors out of 9 beagle dogs (both dogs had nuclear sclerosis) (Hernandez et al. 2016)	no correlation between the age and refractive error was found (de Oliveira et al. 2020).	hyperopia was diagnosed among 38 geriatric mixed breed dogs
Myopia revealed in older dogs with nuclear sclerosis of crystalline lenses	study performed on 240 various breed dogs (Murphy et al. 1992)	2 seniors out of 9 beagle dogs, both dogs had nuclear sclerosis (Hernandez et al. 2016)	none of the dogs in our study had nuclear sclerosis
The study revealed that the degree of myopia develops with age	geriatric dogs had the highest refractive error (–3 D) (Hernandez et al. 2016)	degree of myopia increased with increasing age across all breeds; 1 440 dogs of 90 different breeds (Kubai et al. 2008)	geriatric dogs had the highest value of refractive error (myopia: –2.75 D; hyperopia: 2.5 D)

Studies analysing the refractive status in human myopia are commonly diagnosed in adults, while more older people, regardless of the geographic location, were diagnosed with hyperopia (Lavery et al. 1988; Attebo et al. 1999; Yekta et al. 2009; Cumberland et al. 2015; Nowak et al. 2018). Myopia is associated with a younger age, female sex and the presence of cataracts (Nowak et al. 2018). A report on an investigation on refractive errors in the United Kingdom revealed that the frequency of hyperopia increases with age (7% at 40–44 years) to a level of 46% at the age of 65–69 years (Cumberland et al. 2015). Among 1 367 people from Iran aged 55 to over 80, myopia was diagnosed in 27.2% and hyperopia in 51.6% (Yekta et al. 2009). In our study conducted on 99 dogs, the prevalence of hyperopia was diagnosed among the ametropic eyes in the senior and geriatric dogs. According to our analysis, hyperopia was diagnosed in 47% of the geriatric eye globes.

In a publication from 2020, which focused on the ophthalmic health and refractive state of 62 working dogs, only 12 (19%) were considered emmetropic. Among the ametropic dogs (81%), myopia was diagnosed in 40 (65%) and hyperopia was diagnosed in 10 (16%), while only three dogs (0.05%) were anisometropic. No correlation between the age and the refractive error was found (de Oliveira et al. 2020). In our study, there were nine anisometropic dogs (9%). Emmetropia was diagnosed in 20% of the dogs. The predominance of far-sightedness over short-sightedness was observed in dogs of different ages. No statistically significant correlation between the adult, senior and geriatric dogs was found between the dogs with myopia, while the geriatric dogs had significantly higher hyperopia than the senior dogs (*P*-value 0.02).

In general, the main goal of pupil dilation is the elimination of the error related to the accommodation of the eye. Hernandez et al. (2016) and Groth et al. (2013) conducted studies using a handheld autorefractor to measure the refractive status of dogs. In all these projects, the method was found to be applicable and useful. The research performed with the use of a skiascopic ruler and a Welch Allyn SureSight^TM^ autorefractometer found agreement and no statistically significant differences between the results were obtained using both techniques without dilating the pupil (Groth et al. 2013). Although the comparison of a handheld autorefractor versus streak retinoscopy in dogs showing non-cycloplegic autorefraction had good agreement with the streak retinoscopy, a study conducted by Itoh et al. (2011) revealed the autorefractor, as a device, possibly affected with accommodation and recommended application of the cycloplegic therapy. Differences greater than 0.5 D between the results occurred in 54.3% of the eyes without paralysis accommodation and in 47.8% of the eyes after its elimination (Itoh et al. 2011). Refraction studies performed by the authors with the use of a Retinomax 3 autorefractor were easy to perform and did not require the use of pupil dilators. Additionally, the method was convenient for owners as they did not have to be warned about the precautions and side effects of cycloplegics.

In conclusion, it could be stated that regarding different ages, ametropia is a common refractive state for dogs. Hyperopia is a more common refractive error in older dogs than myopia. Both myopia and hyperopia are more advanced with age.
